# Whole-genome sequencing and disease-gene detection

**DOI:** 10.1186/1753-6561-6-S6-O7

**Published:** 2012-10-01

**Authors:** Lynn B Jorde

**Affiliations:** 1 Department of Human Genetics, University of Utah School of Medicine, UT, USA

## 

Whole-genome sequencing (WGS) offers unique opportunities to identify rare variants that cause disease. We have developed a new software tool, VAAST (Variant Annotation, Analysis and Search Tool) that permits the identification of specific disease-causing mutations in WGS data. VAAST unambiguously identifies two disease-causing mutations in a family quartet in which both offspring have autosomal recessive primary ciliary dyskinesia and Miller syndrome. In addition, VAAST has identified a new X-linked progeria-like syndrome (Ogden syndrome) using exome data from two unrelated families. The mutation occurs in *NAA10*, which encodes an N-acetyltransferase needed for N-terminal acetylation of proteins. Functional studies demonstrate that the mutation causes a loss of function, and a genetic test has been developed for Ogden syndrome. We have also used VAAST to identify *GATA4* as the cause of cardiac septal defects in a single four-generation pedigree. Using the Utah Population Database, we have identified a large multigenerational pedigree in which VAAST, combined with analysis of shared genome segments, identifies a new locus for Crohn disease. Finally, we present an application of VAAST in the identification of *ATP1A3* as a causal gene for alternating hemiplegia of childhood.

